# The “Namaste Sign”—A Novel Coronal MRI Marker for the Evaluation of Anterior Cruciate Ligament Integrity

**DOI:** 10.3390/jcm15176699

**Published:** 2026-08-29

**Authors:** Shashank Chapala, Rajesh Botchu, Hasaam Uldin, Meili Jade Temsin Leung Chew, Kapil Shirodkar, Sang Bum Lee, Anoop Venkatapura Bylaswamy, Avneesh Chhabra

**Affiliations:** 1 Department of Radiology, AIG Hospital, Hyderabad 500032, India; 2 Department of Musculoskeletal Radiology, Royal Orthopaedic Hospital, Bristol Road South, Northfield, Birmingham B31 2AP, UK; 3 Sir Seewoosagur Ramgoolam Medical College, Dalgotiere 80203, Mauritius; 4 Department of Musculoskeletal Radiology, Royal Lancaster Infirmary, Lancaster LA1 4RP, UK; 5 Department of Radiology, UT Southwestern Medical Center, Dallas, TX 75390, USA; sangbum.lee@utsouthwestern.edu (S.B.L.); anoopvbusmle@gmail.com (A.V.B.);

**Keywords:** anterior cruciate ligament, magnetic resonance imaging, coronal plane, partial ACL tear, Namaste sign, mucoid degeneration

## Abstract

Magnetic resonance imaging (MRI) is universally recognized as the gold standard for evaluating anterior cruciate ligament (ACL) injuries. However, differentiating partial tears from complete ruptures using the standard sagittal plane remains a diagnostic challenge. The oblique coronal orientation of the ACL often leads to volume averaging artifacts and inaccurate interpretations. While axial imaging offers a favorable alternative, distal tears near the tibial attachment are frequently missed. This review synthesizes current concepts in the anatomical and radiological evaluation of the ACL and introduces the “Namaste sign,” a novel and proposed preliminary imaging marker observed on coronal MRI to help assess ACL integrity. Grounded in the distinct native double-bundle architecture of the ACL, the Namaste sign utilizes the coronal plane to evaluate the transverse relationship and physiological tension of the anteromedial and posterolateral bundles. In a normal knee, these bundles converge symmetrically from their distinct tibial footprints and maintain tight contact with the lateral femoral condyle, mimicking hands pressed together in a traditional “Namaste” gesture. This article details the anatomical basis of this sign, provides a systematic method for its identification, and illustrates how morphological variations such as “broken hands,” “absent hands,” “absent proximal hug,” or “separation of hands” may correspond to specific ACL lesions. As a preliminary conceptual marker, the Namaste sign is intended to serve as an educational adjunct to combined three-plane evaluation rather than a standalone diagnostic criterion. Notably, this sign is not applicable to postoperative ACL reconstructions. A preliminary pilot study of 50 cases utilizing thin-slice MRI (≤3 mm) demonstrated nearly 100% visibility of the sign, 98% diagnostic accuracy, and almost perfect inter-observer agreement between two radiologists with more than 10 years of experience (Cohen’s kappa = 0.92). However, future diagnostic studies with rigorous arthroscopic reference standards are required to establish its clinical performance and reliability in larger cohorts.

## 1. Introduction

### 1.1. Epidemiology and the Economic Burden of ACL Injuries

Anterior cruciate ligament (ACL) tears are among the most prevalent and debilitating musculoskeletal injuries, particularly in young and active populations [1], with an annual incidence in the United States estimated at over 200,000 [2]. In addition to the immediate athletic impact, there are many long-term consequences of an injured ACL, and despite the high frequency of these injuries, achieving a precise diagnosis remains a significant clinical challenge. Differentiating among a partial tear, a functionally incompetent intact-appearing ligament, and a complete rupture is difficult based on physical examination alone, which is often confounded by acute hemarthrosis and muscle guarding by the patient [3].

### 1.2. The Danger of Missed and Mischaracterized Tears

Despite the high frequency of these injuries, achieving a precise diagnosis remains a significant clinical challenge. Differentiating among a partial tear, a functionally incompetent intact-appearing ligament, and a complete rupture is notoriously difficult based on physical examination alone, which is often confounded by acute hemarthrosis and muscle guarding by the patient [3]. When an ACL injury, particularly a partial or functionally incompetent tear (where the ligament appears morphologically continuous but is biomechanically stretched and insufficient), is missed, the joint is subjected to chronic, translational–rotational kinematic instability. Over time, untreated ACL injury causes persistent knee instability, secondary meniscal tear, chondral injury, and significantly accelerated post-traumatic osteoarthritis, severely impairing overall knee function and the patient’s quality of life [4,5].

### 1.3. MRI Evaluation and Limitations of Standard MRI Assessment

Magnetic Resonance Imaging (MRI) is universally recognized as the gold standard for the non-invasive evaluation of the ACL [6]. However, traditional diagnostic paradigms rely heavily on the sagittal plane due to quick assessment protocols and conventional interpretation training. Because the native ACL is oriented obliquely in the coronal plane and does not lie in a true orthogonal body sagittal plane, standard sagittal slices are highly susceptible to volume averaging artifacts [7]. A partially torn, elongated, or mucoid-degenerated ACL may falsely appear continuous on a sagittal sequence if bridging fibers, scar tissue, or the surrounding synovial sheath remain intact. Alternatively, an intact ACL can appear injured due to partial voluming effects. Furthermore, while the axial plane is excellent for evaluating the femoral notch, it frequently fails to adequately capture the ligament’s tibial attachments and the complex mid-substance continuity [8].

### 1.4. Why Another MRI Sign Is Needed?

In the high-stakes environment of orthopedic imaging, diagnostic confidence and accuracy are paramount. Failure to recognize partial tears or completely detached ligaments that flop into a pseudointact position on sagittal MRI can lead to failed conservative management and early-onset osteoarthritis. While established signs on the coronal plane already exist in the literature, such as the “empty lateral wall sign” or visually undulating and wavy ligament contours, these are primarily indicative of complete midsubstance or proximal ruptures and may lack the subtlety to characterize individual bundle tension.

The coronal plane fills this critical diagnostic gap by providing a direct transverse perspective that overcomes sagittal volume averaging. However, in routine clinical practice, a combined three-plane evaluation (axial, coronal, and sagittal) is mandated to achieve maximum diagnostic accuracy. No single imaging sign on an isolated plane can achieve absolute diagnostic correctness. The “Namaste Sign” equips radiologists, mid-levels, and orthopedic surgeons with a simple, educational, and proposed cross-check MRI feature. By specifically evaluating the presence of the proximal femoral “hug” (attachment with tension) and the “Namaste” configuration (convergence of two bundles), clinicians can improve the characterization of both partial and full-thickness tears [9]. Therefore, this novel sign must be viewed as a complementary tool rather than a sole diagnostic criterion.

#### Literature Search Strategy and Narrative Review Methodology

This manuscript is structured as a conceptual and narrative pictorial review. A comprehensive literature search was conducted using PubMed and Google Scholar, identifying articles related to “Anterior Cruciate Ligament,” “MRI of ACL”, “Coronal plane MRI Knee”, “Anatomy of ACL”, and “Partial Tears of ACL” As a conceptual introduction of a proposed imaging sign, this review does not employ strict systematic inclusion/exclusion criteria or formal quality assessment of primary data, which is standard practice for narrative educational pictorial essays.

## 2. Anatomy and Biomechanics of ACL

The anterior cruciate ligament (ACL) is made of densely packed collagen fibers connecting the femur to the tibia. It consists of two bundles: the anteromedial and posterolateral. Both bundles originate from the femur at the posterior aspect of the inner surface of the lateral femoral condyle [10,11]. The femoral origin of the ACL is oval-shaped, with the anteromedial (AM) bundle’s center positioned more superiorly near the “over-the-top” location and the posterolateral (PL) bundle’s center located closer to the anterior and inferior cartilage edge.

To fully appreciate the radiological appearance of the ACL in the coronal plane, a precise understanding of its native double-bundle architecture and osseous attachments is requisite. The broad femoral footprint is compartmentalized by distinct bony landmarks that dictate the highly specific origins of its two constituent bundles [12,13]. The most prominent of these landmarks is the lateral intercondylar ridge, classically referred to in the arthroscopic literature as the “resident’s ridge.” This ridge runs longitudinally and forms the definitive anterior boundary of the entire ACL attachment site [14]. Positioned posterior to the resident’s ridge is the lateral bifurcate ridge, a more subtle osseous prominence oriented roughly perpendicular to the intercondylar ridge.

The bifurcate ridge distinctly divides the femoral footprint into two separate origin sites [15], with the anteromedial (AM) bundle originating more proximally and posteriorly than the posterolateral (PL) bundle, which attaches more distally and anteriorly within the intercondylar notch. On MRI, these ridges are not clearly identified, but the femoral attachment is observed as a single oval-shaped hypointense band of ligament tightly hugging the femoral condyle.

The long axis of the ACL is angled approximately 26 ± 6 degrees forward from the vertical [10,16], and its curvature aligns with the posterior articular edge of the lateral femoral condyle [10]. As the ACL approaches its tibial insertion anterior and lateral to the medial intercondylar tubercle, its fibers spread out widely with an insertion site that is broad in the anteroposterior direction [10,11,17,18]. Some ACL fibers extend further anteriorly, passing under the transverse meniscal ligament, and a few may merge with the anterior attachment of the anterior horn of the lateral meniscus and the posterior attachment of its posterior horn. Notably, the tibial and femoral insertions of the ACL are more than 3.5 times larger than the midsubstance of the ligament [18].

As the knee joint is flexed, the femoral attachment of the ACL assumes a more horizontal orientation and the anteromedial bundle is more strongly tensioned and taut, while the posterolateral bundle is lax. Conversely, when the knee joint is extended, the posterolateral bundle is taut, and the anteromedial bundle becomes lax [18]. This alternating tension ensures that the ACL stays taut, stabilizing the knee throughout its range of motion. Both bundles resist anterior tibial translation depending on knee position. Additionally, the posterolateral bundle plays a key role in maintaining rotational stability when the knee is nearly extended [19]. Both the anteromedial and posterolateral bundles act synergistically to stabilize the knee joint, sharing a critical role in providing rotational constraint in response to combined rotatory loads [20]. Furthermore, the recent literature emphasizes that ACL injury and subsequent recovery are region-specific. Skeletal muscles and ligaments are not spatially uniform; therefore, the healing capacity and biomechanical responses may vary significantly along the different lengths and regions of the tissue [21]. As outlined by Sahinis et al., intrinsic regional differences in vascularity, cellularity, and extracellular matrix composition mean that the proximal, midsubstance, and distal segments of a ligament respond differently to mechanical stress and injury [21]. Acknowledging these regional variations, where the femoral footprint (“the hug”) biologically differs from the tibial footprint, is critical when interpreting distinct injury patterns and their respective healing potentials.

In general, the AM bundle is most important and contributes 80% of the strength of the ACL. In flexion, the anteromedial bundle primarily prevents anterior tibial translation, and its deficiency is clinically assessed with the anterior drawer test at 90 degrees of flexion. When the knee is in near extension, the posterolateral bundle takes over as the main restraint, and its deficiency can be detected by the Lachman test or the pivot shift test.

## 3. Anatomical Basis of the “Namaste Sign” on MRI

The conceptual framework of the “Namaste sign” is grounded in the native double-bundle anatomy of the ACL, which acts synergistically. As visualized on coronal 2D or 3D MRI sequences, progressing from anterior to posterior, the bundles initially appear distinct at the level of the proximal medial collateral ligament (MCL). As the imaging plane moves posteriorly toward the femoral insertion, the healthy ACL demonstrates a characteristic convergence.

The term “Namaste” aptly describes this morphological union, where the AM and PL bundles (the two hands) press together symmetrically. Furthermore, the sign incorporates the crucial concept of “hugging,” where the united bundles closely abut the medial wall of the lateral femoral condyle. This “hug” represents physiological tension. In the setting of a tear, this tension is lost; the bundles separate from one another or detach from the condylar wall, allowing synovial fluid to interpose, a phenomenon we describe as the “missing Namaste sign.”

### Overcoming MRI Limitations: Coronal Plane Assessment and 3D Imaging

**Figure 1:**
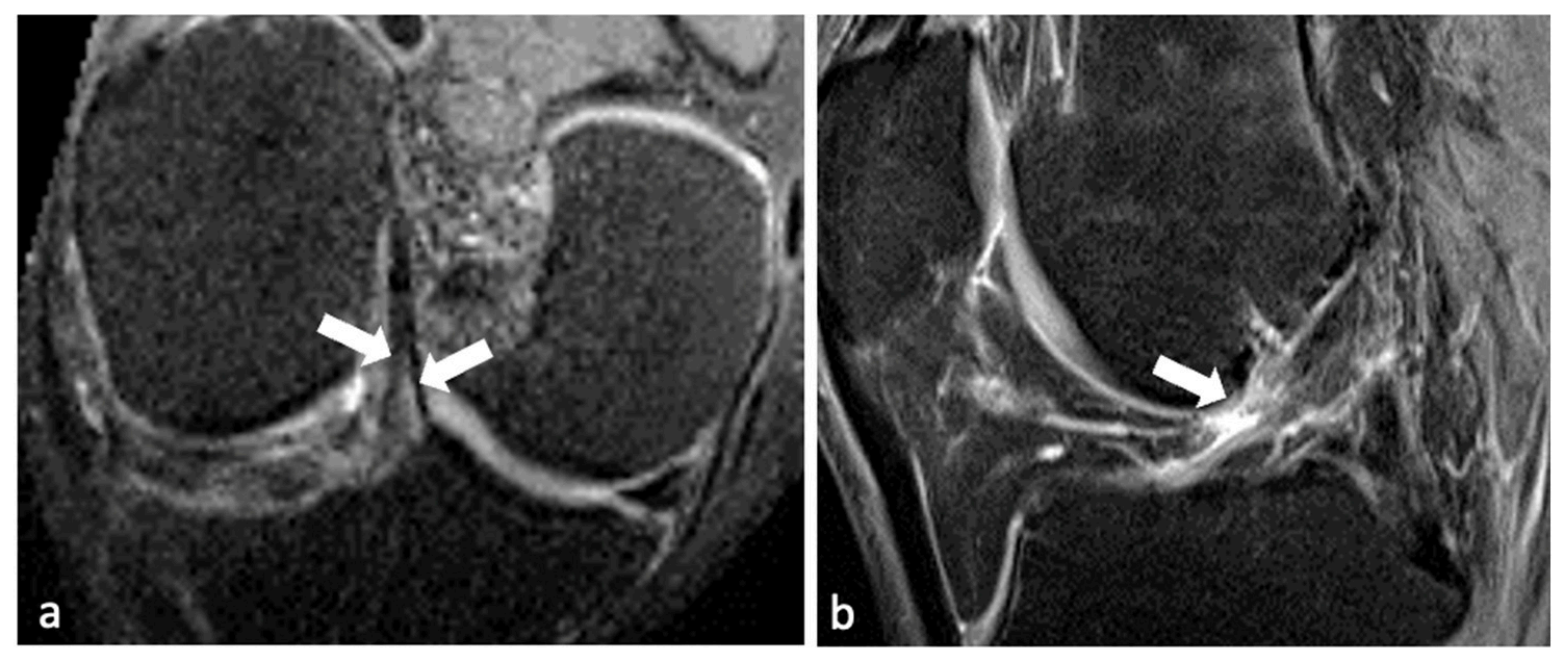
Normal ACL on 3D PDFS (**a**); partial voluming mimicking tear on 2D PDFS (**b**) (3D ACL-specific plane reconstruction). Arrows denote the normal ACL on the 3D PDFS image (**a**) and the area of partial voluming mimicking a tear on the standard 2D PDFS image (**b**).

Due to the established limitations of orthogonal imaging [7], acquiring standard MRI sequences with the knee in external rotation aligns the ACL more parallel to the sagittal imaging plane, allowing for better visualization along its entire length [22]. However, this requires an additional imaging sequence, which is not universally obtained. With the frequent use of 3D isotropic volume MR imaging, one can obtain high-resolution multiplanar reconstructions (MPR) with ACL-specific plane renderings [23,24]. This enables the radiologist to format images in the true oblique plane of the ACL post-acquisition, mitigating technologist planning mishaps and improving diagnostic accuracy for both primary bundle continuity and partial tears [23] (Figure 1).

**Figure 2:**
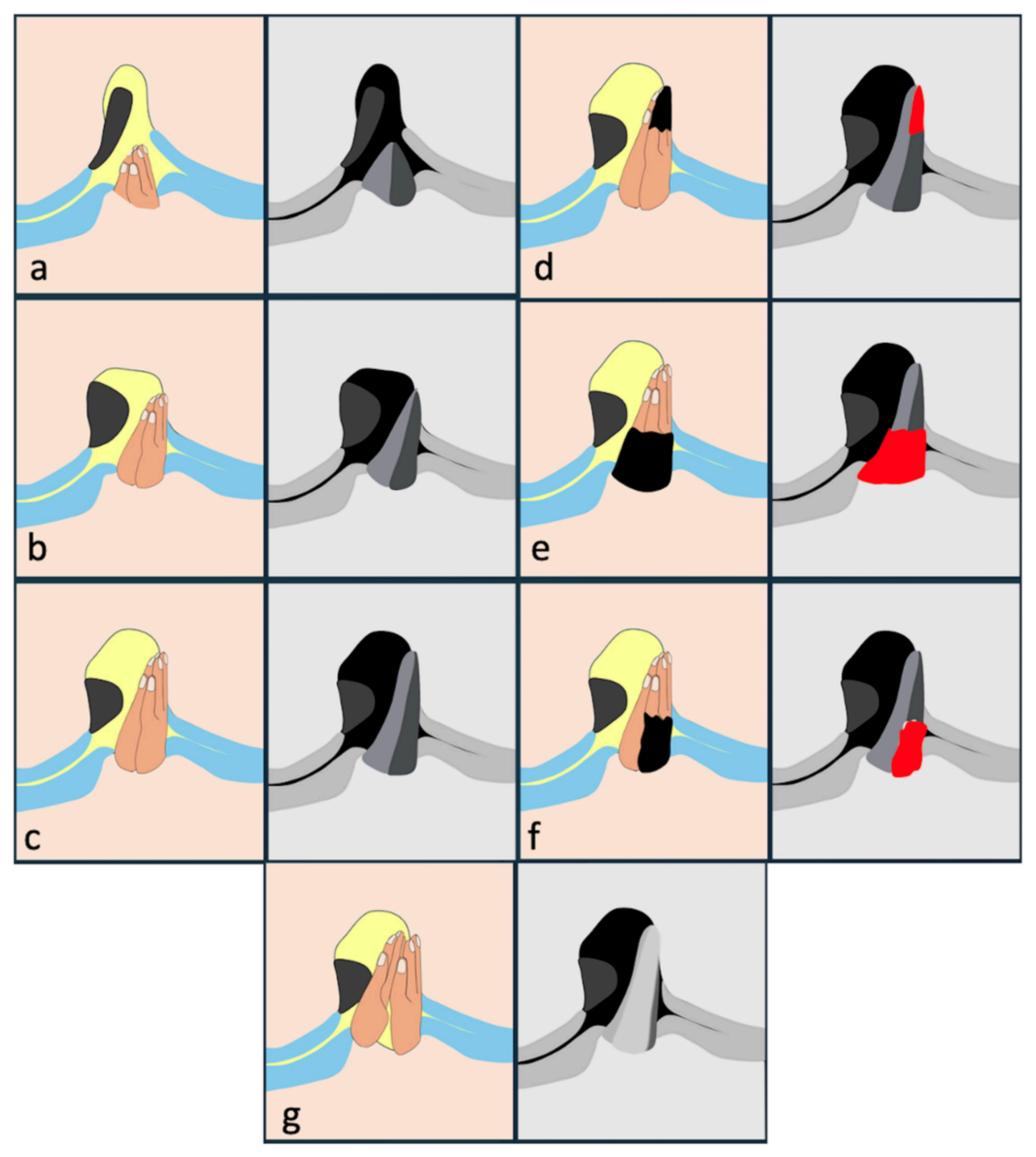
Illustration of the Namaste sign (in coronal plane): Intact Namaste in normal ACL (**a**–**c**); “absent proximal hug” in partial thickness tear of posterolateral bundle at femoral attachment (**d**); “broken hands” in tear of both bundles at tibial attachment (**e**); “broken hand” in tear of posterolateral bundle of ACL (**f**); and “separation of hands” in mucoid degeneration of ACL (**g**).

The coronal plane serves as an invaluable, yet often underutilized, asset for evaluating the transverse relationship of the ACL’s double-bundle architecture [25]. This article introduces the “Namaste sign,” a novel proposed morphological marker observed on standard coronal T2-weighted MRI sequences. By tracking the natural, physiological convergence of the AM and PL bundles as they move posteriorly to “hug” the lateral femoral condyle, this sign allows for a direct visual assessment of ligamentous tension (Figure 2).

Namaste is an ancient Sanskrit word, a combination of “namah” plus “te”, meaning that the divinity in a person doing the Namaste gesture respects the divinity in the other person. The gesture itself denotes two folded hands coming together in the midportion as a single compact-appearing top with two distinct bases. The presence of this sign confirms the integrity of the ACL, and the absence of any portion of the “Namaste” morphology indicates ACL lesions.

## 4. How to Identify the “Namaste Sign”?

**Figure 3:**
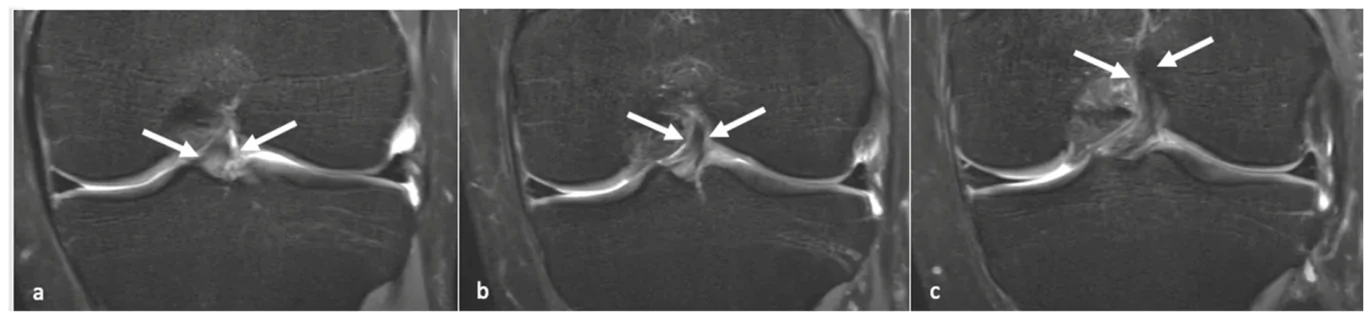
PDFS coronal (**a**–**c**): Three steps to assess ACL Namaste. The arrows point to the intact anteromedial and posterolateral bundles converging and pressing against the femoral condyle to form the complete “Namaste” shape.

This sign is best appreciated on sequential coronal fluid-sensitive 2D MRI sequences in three slices, as an alternative to a single-slice depiction of an ACL-plane MRI image or an ACL-plane reconstructed image from 3D isotropic volume scanning. As one scrolls from anterior to posterior on coronal images, there are three steps to identify the sign (Figure 3).

Step 1: (Anterior view interrogation—“bottom of the hands”) As one evaluates the coronal slices, the tibial attachment of the ACL is seen as two distinct and intact bundles (the bases of two hands doing Namaste), where the medial collateral ligament (MCL) is clearly visible. Depending upon the slice thickness, these bundles may be seen distinctly on one to two slices for 3–4 mm slice acquisitions, or several slices if sub-1.0 mm 3D MRI slices have been reconstructed.

Step 2: Mid-posterior scroll (The “joining hands”) Upon scrolling posteriorly at the level of the posterior oblique ligament, the two separate bundles begin to converge and should remain intact.

Step 3: Most posterior view (The “hug”) Upon scrolling further posteriorly, these joined bundles show a tight, conjoint oval-shaped attachment at the medial wall of the lateral femoral condyle as a tight hug, mimicking the closely apposed fingertips of the “Namaste sign.”

## 5. Interpretation: Normal ACL vs. Lesions

**Figure 4:**
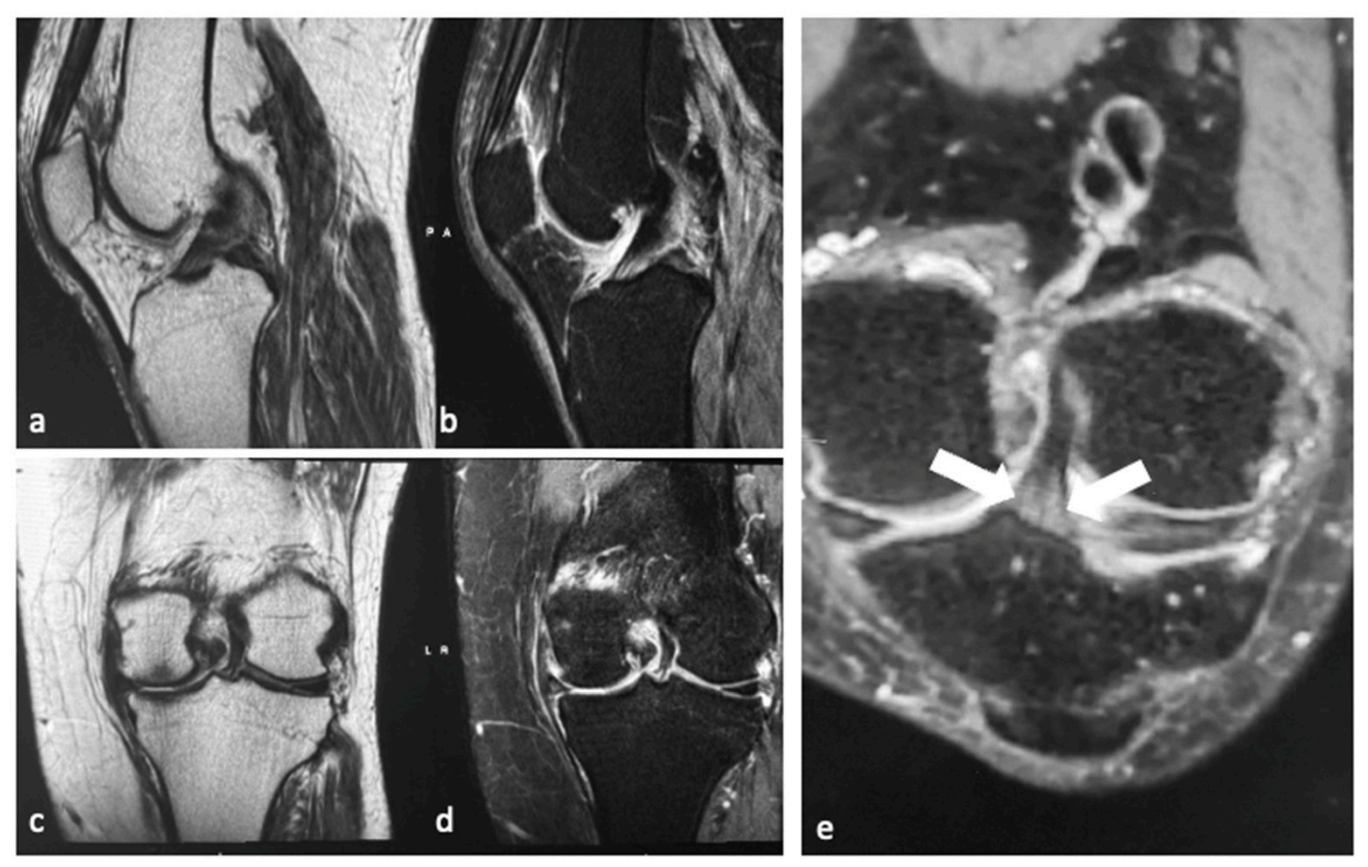
PD (**a**), PDFS (**b**) sagittal, PD (**c**), PDFS (**d**) coronal, and 3D coronal PDFS (**e**) arrow showing normal ACL Namaste configuration.

The “Namaste sign” can be considered a critical check for continuity and tension of the ACL. If the ACL is intact, the bundles remain taut, continuous, and pressed together and against the femoral condyle, forming a good “Namaste Sign” morphology on MRI (Figure 4).

In the event of an ACL lesion—commonly an individual PL bundle tear, both bundles tear, or mucoid degeneration—the hands of Namaste may be broken at different places at the site of the tear or spread apart, as with mucoid degeneration.

## 6. Interpretation and Region-Specific Clinical Implications

**Figure 5:**
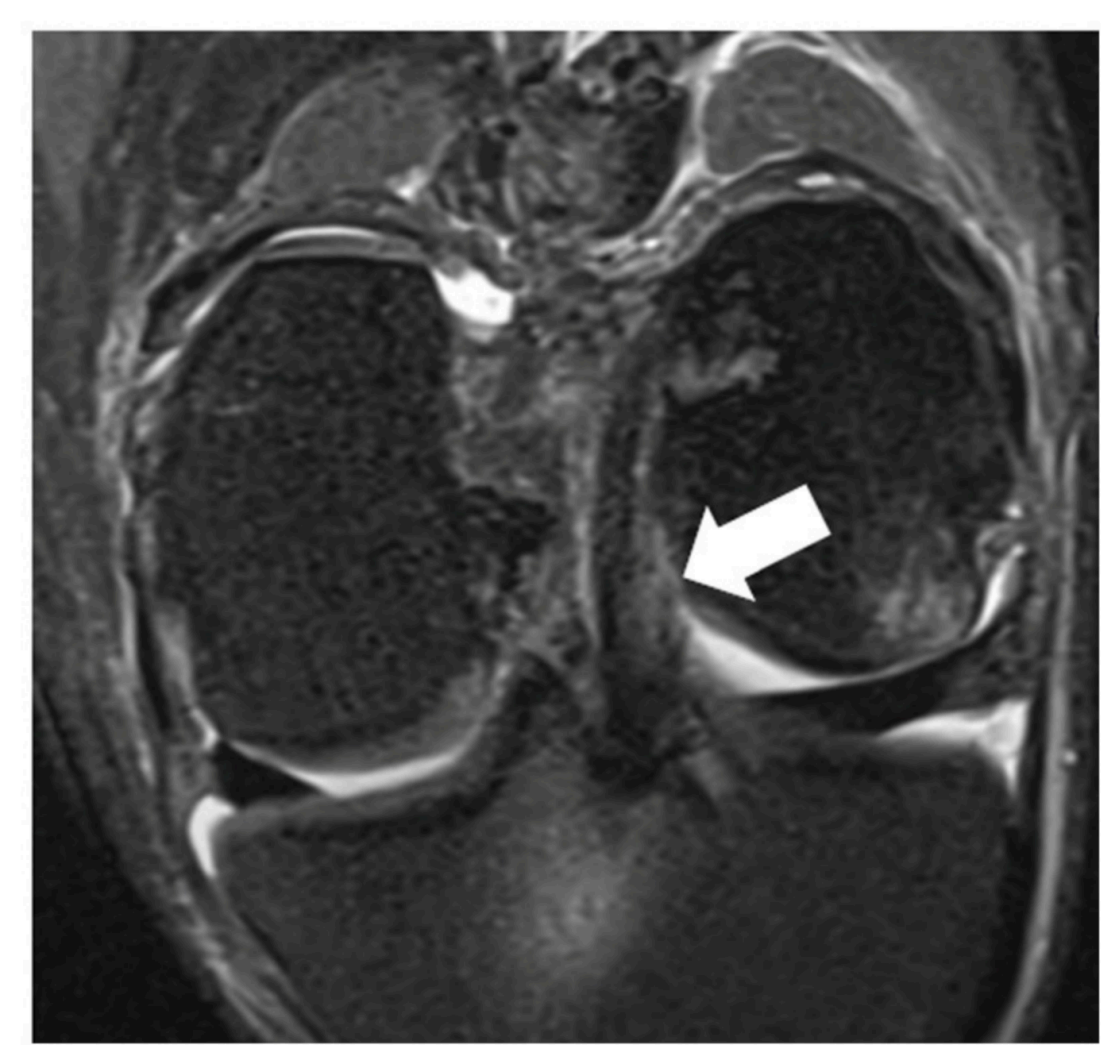
PDFS coronal showing tear of the posterolateral band of ACL (arrow).

**Figure 6:**
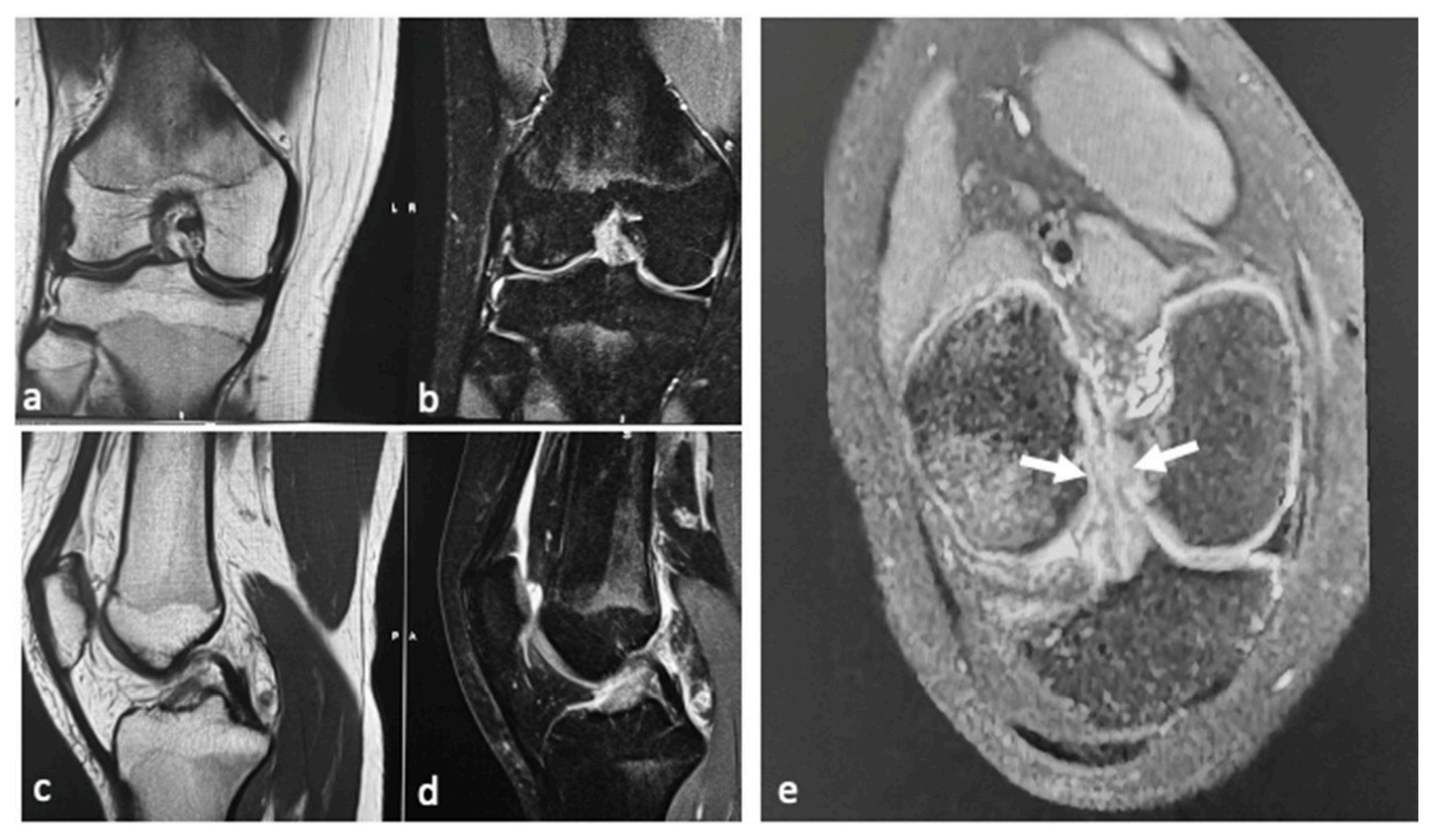
PD (**a**), PDFS (**b**) coronal; PD (**c**), PDFS (**d**) sagittal; and 3d coronal PDFS (**e**) showing full-thickness tear of the midsubstance of the ACL (arrow)—Absent fingers of Hand—2D and 3D.

**Figure 7:**
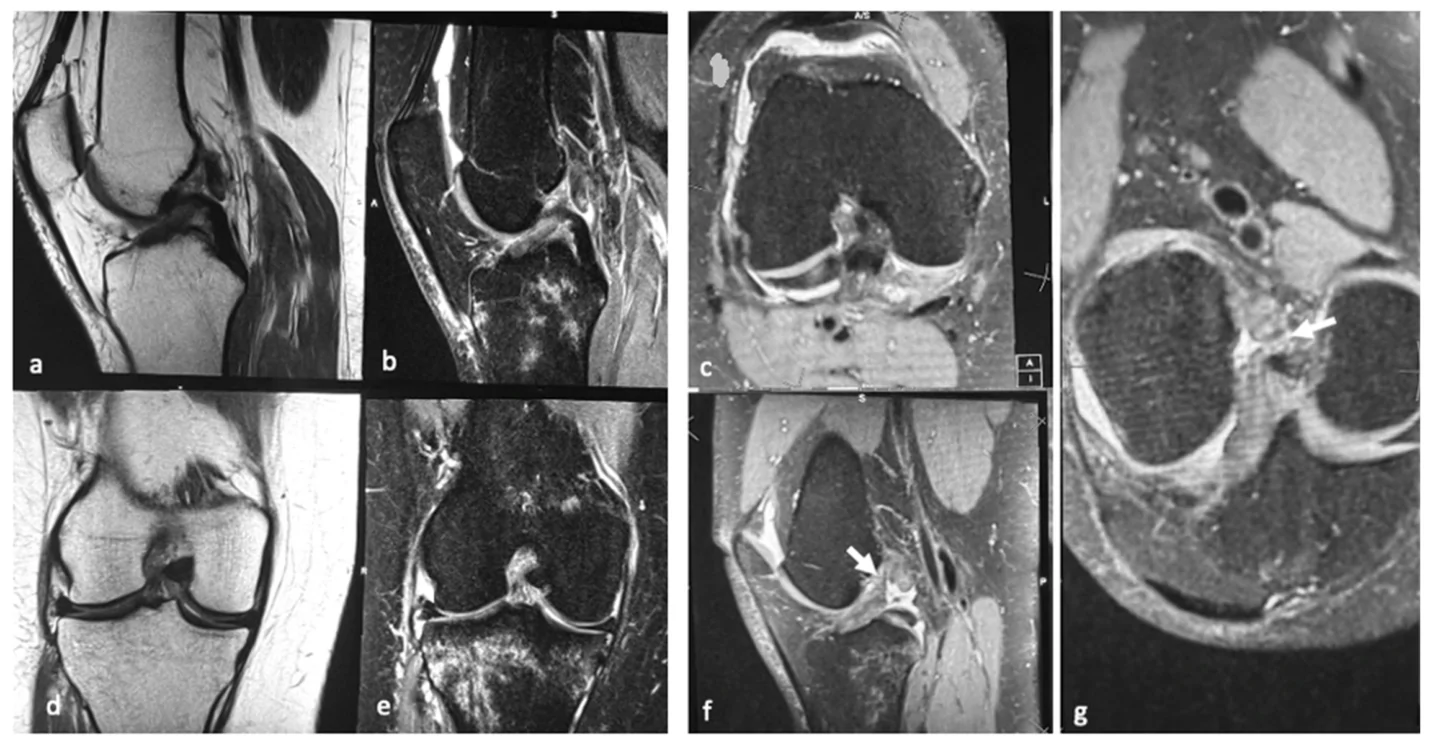
PD sag (**a**,**d**), PDFS sag (**b**,**e**) and axial (**c**,**f**) and 3D (**g**)—Two different cases showing full-thickness tear of the ACL at its femoral attachment (arrow)—absent Hug configuration of bundles of ACL.

**Figure 8:**
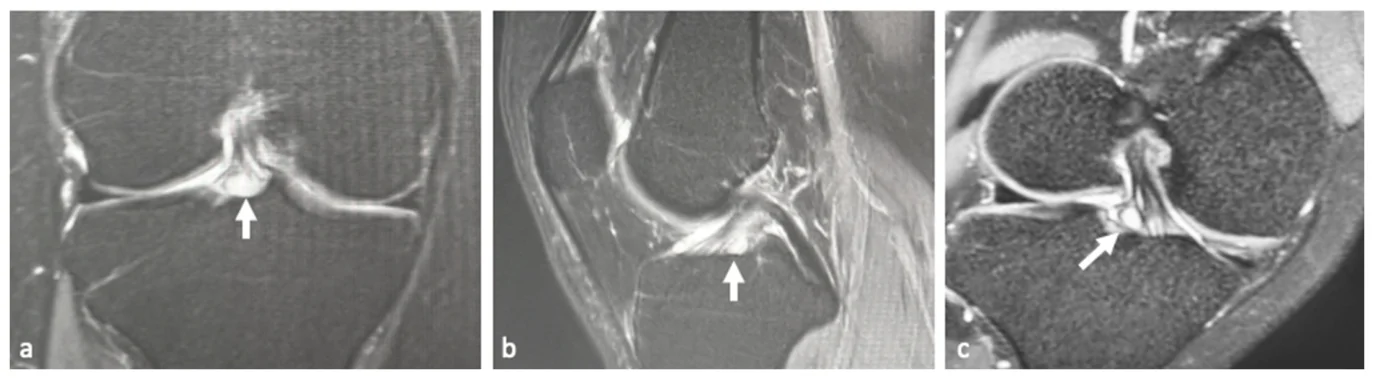
PDFS coronal (**a**), sagittal (**b**) and 3D (**c**) shows broken one hand of Namaste in keeping with tear involving the posterolateral bundle of ACL with ganglion (arrow).

**Figure 9:**
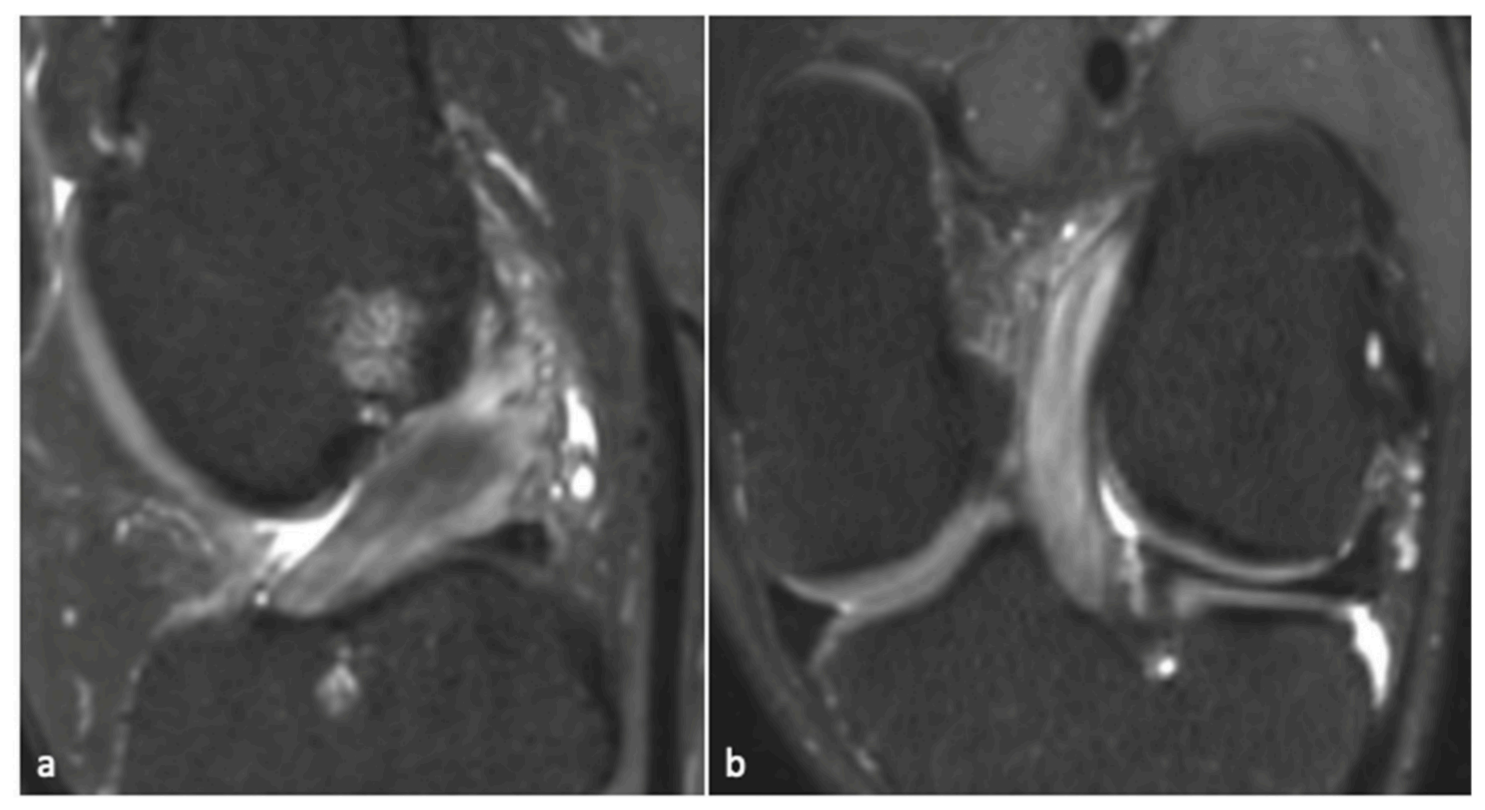
PDFS sagittal (**a**) and coronal (**b**) showing mucoid degeneration of ACL mucoid—separated hands of Namaste.

To better understand the proposed Namaste sign, the morphological variations can be organized into a region-specific framework. Native ACL biomechanics dictate that the anteromedial (AM) bundle primarily restricts anterior tibial translation, particularly in flexion, while the posterolateral (PL) bundle is crucial for resisting rotatory loads, especially near full extension [20] (Figure 5, Figure 6, Figure 7, Figure 8 and Figure 9).

1. Tibial Attachment Abnormalities (“Bottom of the Hands”):

The “broken hand(s) of Namaste”, representing a partial tear or isolated rupture of either the AM or PL bundle at or near the tibial footprint, correlates directly with selective translational or rotational instability, respectively [26]. In cases of chronic ACL deficiency, characterized on coronal MRI by “absent hands and Namaste”, the knee suffers from gross multidirectional instability [27].

2. Midsubstance Abnormalities (“Joining Hands”):

The “separation of hands” scenario presents a different clinical challenge. This is characterized by complex fluid interposed between mostly intact ACL fibers, visibly pushing the AM and PL bundles apart on coronal views in the midsubstance. This appearance is highly compatible with mucoid degeneration and/or an intraligamentous ganglion cyst [28,29]. A periligamentous notch ganglion or intraosseous ganglion may be present. Clinically, this “separation” manifests not as joint instability, but as a mechanical block. The thickened, swollen ligament impinges upon the intercondylar notch, frequently causing localized pain and a locking sensation in terminal extension [28].

3. Femoral-Sided Tears (“The Hug”):

An “absent proximal hug,” characterized by a partial or complete proximal ACL tear at the femoral attachment with a fluid gap between the ligament and the condyle, highlights a crucial diagnostic trap. In this scenario, the ligament fibers may appear structurally continuous on sagittal imaging; however, the coronal loss of the “hug” (tension) indicates a functionally incompetent ligament that fails to restrict anterior translation [30].

## 7. Limitations in Post-Operative ACL Evaluation

While the Namaste sign is proposed to be valuable in the native knee, its diagnostic utility does not reliably extend to the postoperative setting. ACL reconstruction (ACLR) utilizes a variety of graft types (autograft, allograft) and surgical techniques. Single-bundle reconstruction remains the most widely performed technique globally [31]. Naturally, a single-bundle graft lacks the biphasic architecture required to form the two “hands” of the Namaste configuration.

**Figure 10:**
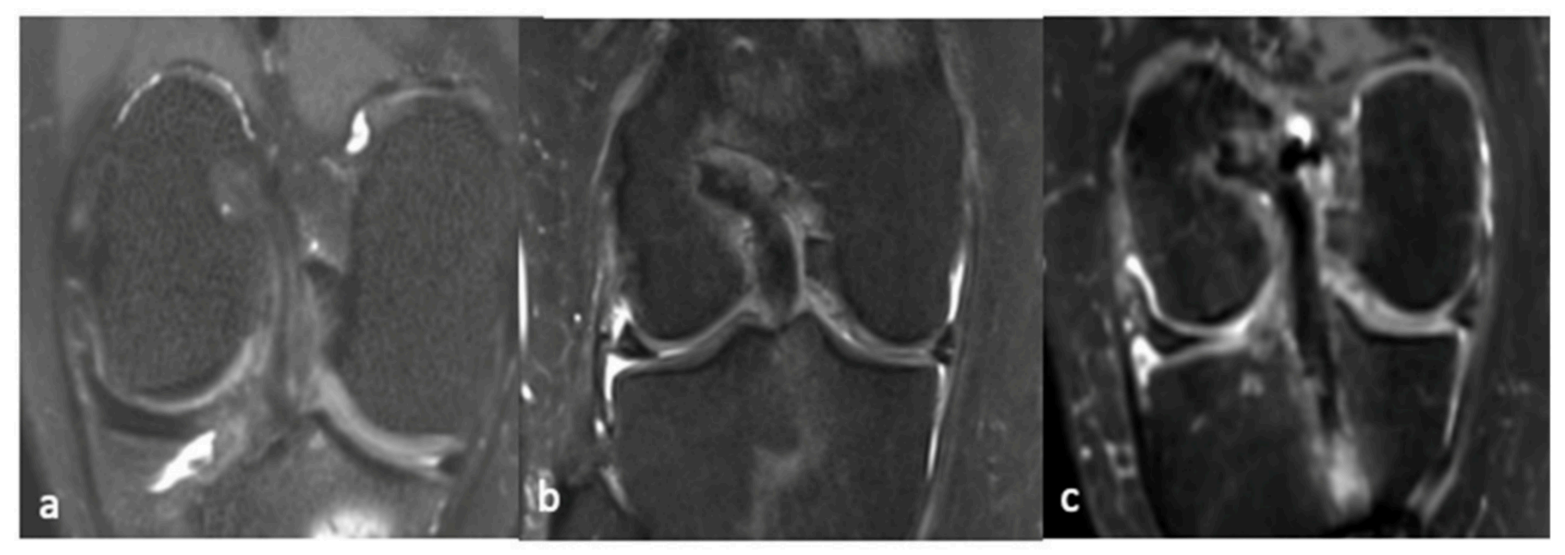
Two cases of ACL reconstruction using quadriceps autograft: PDFS coronal 3D; (**a**) shows the Namaste sign is present but unclear, especially the irregular but intact AM bundle. Second case (coronal PDFS (**b**) and 3D (**c**)) 6 months post ACL reconstruction surgery, in the process of ligamentization/graft maturation.

Even in the context of anatomic double-bundle reconstruction, which attempts to meticulously restore the native AM and PL footprints, the Namaste sign is generally not applicable due to linear or multilaminar longitudinal arrangements. The distinct convergence and specific condylar “hug” seen in native anatomy are rarely perfectly replicated. Therefore, although coronal MRI remains an excellent and necessary plane for evaluating graft continuity, volume, and tunnel positioning, the specific criteria of the Namaste sign should be reserved exclusively for the evaluation of the primary, unoperated ACL [32,33] (Figure 10).

## 8. Comparison with Established Signs

Current radiological literature emphasizes sagittal signs such as the “Empty notch sign”, “tangent sign”, and discontinuity of the Blumensaat line. While highly specific, these signs can be limited by volume averaging artifacts and can sometimes fail to characterize the extent of bundle-specific injury. The “Namaste sign” is proposed to add value by utilizing the coronal plane to assess the transverse relationship of the bundles. Unlike the “Empty notch sign”, which indicates a complete absence of the ligament, in the authors’ preliminary experience, the “Namaste sign” may assist in identifying subtle partial tears or elongation where gross continuity remains but physiological tension (the “hug”) is lost.

## 9. Clinical Implications and Diagnostic Utility

The clinical utility of the “Namaste sign” lies in its visual simplicity. By anthropomorphizing the anatomy and comparing the converging bundles to hands in this ancient greeting, this sign lowers the learning curve for trainees and non-musculoskeletal radiologists. Furthermore, the sign provides a specific checkpoint for “fluid gap” assessment. The presence of hyperintense fluid signal separating the ACL bundles from the lateral femoral condyle (the loss of the hug) serves as a high-contrast marker for pathology. This may reduce false negatives in cases where the ACL appears structurally continuous on sagittal views but is functionally incompetent or detached.

Furthermore, the morphological patterns of the Namaste sign appear to visually parallel the established Sherman classification of ACL tears, though formal statistical correlation and observer agreement have not yet been calculated. Because this morphological classification is conceptually similar to the Sherman system associating an ‘absent proximal hug’ with Type I/II femoral avulsions, a “separation of hands” with Type III midsubstance tears, and “broken hands” at the tibial insertion with Type IV tears, it may enable greater reporting efficiency. Rather than acting as an additional, burdensome scoring system, the Namaste sign provides a direct visual correlate that could sufficiently replace the need to separately report the older classification, streamlining preoperative staging. This directly benefits surgical decision-making; for example, localizing an isolated “absent proximal hug” may identify ideal candidates for primary ACL footprint repair with cortical button fixation, whereas midsubstance “broken hands” or chronic deficiency would favor traditional graft reconstruction.

To support the clinical applicability of the Namaste sign, we conducted a preliminary pilot study evaluating 50 knee MRI cases acquired with sequences utilizing a slice thickness of 3 mm or less. In this initial cohort, the Namaste sign was identifiable in nearly 100% of optimal thin-slice scans. Preliminary performance metrics yielded a diagnostic accuracy of 98%, sensitivity of 98%, and specificity of 98% for characterizing ACL integrity. Furthermore, inter-observer reliability testing between two musculoskeletal radiologists yielded a Cohen’s kappa coefficient of 0.92, indicating almost perfect agreement.

While these overall preliminary metrics are highly encouraging, the sample size of this initial pilot cohort (*n* = 50) precludes statistically powered subgroup analyses for each specific morphological scenario outlined in Table 1. Validating the sensitivity and specificity of individual variations such as isolated femoral avulsions versus mucoid degeneration remains a primary objective for future, larger-scale investigations. These initial findings underscore the high reproducibility and diagnostic value of the sign when evaluated on appropriate high-resolution sequences.

## 10. Preliminary Pilot Study: Methods and Results

Methods: To support the clinical applicability of the Namaste sign, a preliminary retrospective evaluation was conducted. Case Selection and MRI Protocol: The retrospective cohort consisted of 50 knee MRI cases of patients aged between 14 and 36 years, with a male-to-female ratio of 34:16. Inclusion criteria were patients presenting with acute or chronic knee pain or suspected ACL injury who underwent a knee MRI evaluation that included fluid-sensitive coronal sequences with a slice thickness of 3 mm or less. Exclusion criteria included patients with a history of previous ACL reconstruction or prior extensive knee surgery, advanced osteoarthritis significantly altering native bony anatomy, gross motion artifacts, and incomplete imaging protocols.

Image Interpretation and Blinding: Two musculoskeletal radiologists with over 10 years of experience independently reviewed the MRI sequences. Both readers were completely blinded to the patients’ clinical histories, physical examination findings, and the original official MRI reports. A standard reporting protocol was utilized, whereby each reader independently evaluated the structural continuity of the ACL in standard orthogonal planes and specifically recorded the morphological status of the “Namaste sign” (intact, broken hands, absent hands, absent proximal hug, or separation of hands) based strictly on the coronal sequences.

Reference Standard and Statistical Analysis: Because systematic arthroscopic correlation was not available for this preliminary cohort, the reference standard was based on a final consensus between the clinical presentation and a comprehensive, unblinded multidimensional radiological diagnosis. Statistical analysis was performed using descriptive statistics for patient demographics. The diagnostic accuracy, sensitivity, and specificity of the Namaste sign were calculated against this consensus reference standard. Inter-observer reliability between the two independent readers was evaluated using Cohen’s kappa coefficient, with values > 0.90 considered to indicate almost perfect agreement.

Results: In this preliminary cohort, the Namaste sign was identifiable in nearly 100% of optimal thin-slice scans. When evaluated as an observational marker for ACL integrity, preliminary performance metrics yielded a diagnostic accuracy of 98%, sensitivity of 98%, and specificity of 98%. Furthermore, inter-observer reliability testing between the two radiologists yielded a Cohen’s kappa coefficient of 0.92, indicating almost perfect agreement. A summary of the patient demographics and preliminary diagnostic outcomes is provided in Table 2.

Discussion of Pilot Study: While these initial reproducibility metrics are highly encouraging, the sample size (*n* = 50) precludes statistically powered subgroup analyses. Because arthroscopic confirmation was not systematically applied to the cases in this study, these results should be interpreted cautiously as preliminary feasibility and reproducibility findings rather than a definitive validation of diagnostic performance. Validating the sensitivity and specificity of individual morphological variations against direct surgical visualization remains a primary objective for future, larger-scale investigations.

## 11. Limitations of the Current Study

It is important to acknowledge the limitations of applying this novel sign. As a preliminary conceptual introduction, this narrative review has several inherent methodological limitations. First, while our preliminary pilot study provides initial feasibility metrics (including 98% accuracy, high sensitivity, and near-perfect inter-observer reliability), it is limited by its small sample size (*n* = 50) and a lack of systematic arthroscopic confirmation. Large-scale, multicenter comparative analyses between normal controls and surgically confirmed ACL injury groups are still needed.

Consequently, potential false-positive outcomes (such as severe volume averaging mimicking a tear, or mucoid degeneration mimicking intact but swollen tissue) and false-negative outcomes (such as a scarred, functionally incompetent ligament appearing morphologically continuous and maintaining a pseudohug) have not been formally quantified. This sign is not applicable to reconstructed ligaments, and the reader is advised to check the ligament in all three planes in post-surgery cases [34]. Therefore, the Namaste sign must currently be viewed as a promising, preliminary observational educational tool rather than a proven diagnostic metric. In the future, scientific studies comparing various ACL lesions with controls with arthroscopic correlation will be required to validate the true utility of this novel sign.

## 12. Conclusions

The “Namaste sign” represents an anatomically grounded, conceptual addition to the radiologist’s toolkit for ACL integrity assessment. By focusing on the convergence and condylar approximation of the AM and PL bundles on coronal views, this sign aims to act as a useful adjunct to standard sagittal imaging. However, it must be recognized as an initial descriptive proposal; future prospective studies correlating this MRI sign with arthroscopic findings are strictly necessary to establish its clinical validity, sensitivity, and specificity in broader clinical settings.

## Figures and Tables

**Table 1:** Namaste sign variations and proposed clinical interpretations.

Status	MRI Appearance	Proposed Clinical Interpretation (Pending Validation)
Normal ACL	The “Namaste sign” shape is intact. The two bundles, as viewed from distal to proximal, are intact at the anterior tibial attachment, meet in the midportion, and are flush against the femoral condyle (the “hug”) with no fluid gap.	Findings suggest an intact ACL
ACL injuries	Broken hand(s) of Namaste: one or both bundles missing, broken, not joining together, or distorted in shape	Translational and/or rotational instability
	Absent hands and Namaste: ACL deficiency (one or both bundles missing, broken, or distorted in shape)	Both translational and rotational instability
	Absent proximal hug: partial or complete proximal ACL tear at femoral attachment with a fluid gap between the ACL and the femoral condyle	Can be missed if axial or coronal images are not carefully evaluated. Translational and/or rotational instability
	Separation of hands: complex fluid interposed between mostly intact ACL fibers compatible with mucoid degeneration and/or ganglion of ACL.	Locking in terminal extension.

**Table 2:** Patient Demographics and Preliminary Diagnostic Outcomes.

Parameter	Value
Patient Demographics	
Total Number of Patients (*n*)	50
Age Range (years)	14–36
Sex Distribution (Male:Female)	34:16
Diagnostic Outcomes and Reliability	
Visibility of Namaste Sign on Thin-Slice MRI	~100%
Diagnostic Accuracy	98%
Sensitivity	98%

## Data Availability

Data can be shared on request from the corresponding author.

## References

[1] Spindler K.P., Wright R.W. (2008). Anterior cruciate ligament tear. N. Engl. J. Med..

[2] Sanders T.L., Maradit Kremers H., Bryan A.J., Larson D.R., Dahm D.L., Levy B.A., Stuart M.J., Krych A.J. (2016). Incidence of Anterior Cruciate Ligament Tears and Reconstruction: A 21-Year Population-Based Study. Am. J. Sports Med..

[3] Temponi E.F., de Carvalho Júnior L.H., Sonnery-Cottet B., Chambat P. (2015). Partial tearing of the anterior cruciate ligament: Diagnosis and treatment. Rev. Bras. Ortop..

[4] Luc B., Gribble P.A., Pietrosimone B.G. (2014). Osteoarthritis prevalence following anterior cruciate ligament reconstruction: A systematic review and numbers-needed-to-treat analysis. J. Athl. Train..

[5] Lohmander L.S., Ostenberg A., Englund M., Roos H. (2004). High prevalence of knee osteoarthritis, pain, and functional limitations in female soccer players twelve years after anterior cruciate ligament injury. Arthritis Rheum..

[6] Crawford R., Walley G., Bridgman S., Maffulli N. (2007). Magnetic resonance imaging versus arthroscopy in the diagnosis of knee pathology, concentrating on meniscal lesions and ACL tears: A systematic review. Br. Med. Bull..

[7] Hong S.H., Choi J.Y., Lee G.K., Choi J.A., Chung H.W., Kang H.S. (2003). Grading of anterior cruciate ligament injury. Diagnostic efficacy of oblique coronal magnetic resonance imaging of the knee. J. Comput. Assist. Tomogr..

[8] Steckel H., Starman J.S., Baums M.H., Klinger H.M., Schultz W., Fu F.H. (2007). Anatomy of the anterior cruciate ligament double bundle structure: A macroscopic evaluation. Scand. J. Med. Sci. Sports.

[9] Van Dyck P., De Smet E., Veryser J., Lambrecht V., Gielen J.L., Vanhoenacker F.M., Dossche L., Parizel P.M. (2012). Partial tear of the anterior cruciate ligament of the knee: Injury patterns on MR imaging. Knee Surg. Sports Traumatol. Arthrosc..

[10] Arnoczky S.P. (1983). Anatomy of the anterior cruciate ligament. Clin. Orthop. Relat. Res..

[11] Girgis F.G., Marshall J.L., Monajem A. (1975). The cruciate ligaments of the knee joint. Anatomical, functional and experimental analysis. Clin. Orthop. Relat. Res..

[12] Ferretti M., Ekdahl M., Shen W., Fu F.H. (2007). Osseous landmarks of the femoral attachment of the anterior cruciate ligament: An anatomic study. Arthroscopy.

[13] Nguyen T., Haider S., Tietze D., Xi Y., Thakur U., Shah J., Chhabra A. (2022). Anterior cruciate ligament foot plate anatomy: 3-dimensional and 2-dimensional MRI evaluation with arthroscopy assessment in a subset of patients. Eur. Radiol..

[14] Hutchinson M.R., Ash S.A. (2003). Resident’s ridge: Assessing the cortical thickness of the lateral wall and roof of the intercondylar notch. Arthroscopy.

[15] Giuliani J.R., Kilcoyne K.G., Rue J.P. (2009). Anterior cruciate ligament anatomy: A review of the anteromedial and posterolateral bundles. J. Knee Surg..

[16] Odensten M., Gillquist J. (1985). Functional anatomy of the anterior cruciate ligament and a rationale for reconstruction. J. Bone Jt. Surg. Am..

[17] Smith B.A., Livesay G.A., Woo S.L. (1993). Biology and biomechanics of the anterior cruciate ligament. Clin. Sports Med..

[18] Zantop T., Petersen W., Sekiya J.K., Musahl V., Fu F.H. (2006). Anterior cruciate ligament anatomy and function relating to anatomical reconstruction. Knee Surg. Sports Traumatol. Arthrosc. Off. J. ESSKA.

[19] Dienst M., Burks R.T., Greis P.E. (2002). Anatomy and biomechanics of the anterior cruciate ligament. Orthop. Clin. N. Am..

[20] Zantop T., Herbort M., Raschke M.J., Fu F.H., Petersen W. (2007). The role of the anteromedial and posterolateral bundles of the anterior cruciate ligament in anterior tibial translation and internal rotation. Am. J. Sports Med..

[21] Sahinis C., Amiridis I.G., Kellis E. (2025). Neuromechanical basis of region-specific differences and their implications for sport performance and injury prevention: A narrative review. Eur. J. Appl. Physiol..

[22] Buckwalter K.A., Pennes D.R. (1990). Anterior cruciate ligament: Oblique sagittal MR imaging. Radiology.

[23] Jung J.Y., Yoon Y.C., Kwon J.W., Ahn J.H., Choe B.K. (2009). Diagnosis of internal derangement of the knee at 3.0-T MR imaging: 3D isotropic intermediate-weighted versus 2D sequences. Radiology.

[24] Cheraya G., Chhabra A. (2023). Cruciate and Collateral Ligaments: 2-Dimensional and 3-Dimensional MR Imaging-Aid to Knee Preservation Surgery. Semin. Ultrasound CT MRI.

[25] Starman J.S., Vanbeek C., Armfield D.R., Sahasrabudhe A., Baker C.L., Irrgang J.J., Fu F.H. (2007). Assessment of normal ACL double bundle anatomy in standard viewing planes by magnetic resonance imaging. Knee Surg. Sports Traumatol. Arthrosc..

[26] Petersen W., Zantop T. (2007). Anatomy of the anterior cruciate ligament with regard to its two bundles. Clin. Orthop. Relat. Res..

[27] Louboutin H., Debarge R., Richou J., Selmi T.A.S., Donell S.T., Neyret P., Dubrana F. (2009). Osteoarthritis in patients with anterior cruciate ligament rupture: A review of risk factors. Knee.

[28] Lintz F., Pujol N., Boisrenoult P., Bargoin K., Beaufils P., Dejour D. (2011). Anterior cruciate ligament mucoid degeneration: A review of the literature and management guidelines. Knee Surg. Sports Traumatol. Arthrosc..

[29] Ravi V., Rehman M., Xia S., Chhabra A., Silva F.D. (2025). Association of tibial slope alterations with anterior cruciate ligament (ACL) injury and mucoid degeneration. Skelet. Radiol..

[30] Lee S.H., Yun S.J. (2020). Diagnostic performance of MRI for partial tears of the anterior cruciate ligament: A systematic review and meta-analysis. Eur. Radiol..

[31] Hussein M., van Eck C.F., Cretnik A., Dinevski D., Fu F.H. (2012). Prospective randomized clinical evaluation of conventional single-bundle, anatomic single-bundle, and anatomic double-bundle anterior cruciate ligament reconstruction: 281 cases with 3- to 5-year follow-up. Am. J. Sports Med..

[32] Kulczycka P., Larbi A., Malghem J., Thienpont E., Vande Berg B., Lecouvet F. (2015). Imaging ACL reconstructions and their complications. Diagn. Interv. Imaging.

[33] Thakur U., Gulati V., Shah J., Tietze D., Chhabra A. (2022). Anterior cruciate ligament reconstruction related complications: 2D and 3D high-resolution magnetic resonance imaging evaluation. Skelet. Radiol..

[34] Gnannt R., Chhabra A., Theodoropoulos J.S., Hodler J., Andreisek G. (2011). MR imaging of the postoperative knee. J. Magn. Reson. Imaging.

